# Low Transforming Growth Factor-β Pathway Activity in Cervical Adenocarcinomas

**DOI:** 10.3389/fonc.2022.797453

**Published:** 2022-06-08

**Authors:** Dieuwke L. Marvin, Vivian M. Spaans, Cor D. de Kroon, Roderick C. Slieker, Maryam Khelil, Peter ten Dijke, Laila Ritsma, Ekaterina S. Jordanova

**Affiliations:** ^1^ Oncode Institute and Department of Cell and Chemical Biology, Leiden University Medical Center, Leiden, Netherlands; ^2^ Department of Gynaecology and Obstetrics, Leiden University Medical Center, Leiden, Netherlands; ^3^ Department of Pathology, Leiden University Medical Center, Leiden, Netherlands; ^4^ Department of Cell and Chemical Biology, Leiden University Medical Center, Leiden, Netherlands; ^5^ Department of Epidemiology and Data Science, Amsterdam University Medical Center (UMC), location VU University Medical Center (VUmc), Amsterdam, Netherlands; ^6^ Department of Gynaecology and Obstetrics, Center Gynaecological Oncology Amsterdam, Amsterdam University Medical Center (UMC), location VU University Medical Center (VUmc), Amsterdam, Netherlands; ^7^ Department of Urology, The Netherlands Cancer Institute, Amsterdam, Netherlands

**Keywords:** cervical cancer, adenocarcinoma, squamous cell carcinoma, TGFBR2, SMAD4, transforming growth factor-β (TGF-β)

## Abstract

Cervical cancer is the fourth most common cancer in women worldwide. Squamous cell carcinoma (SCC) and adenocarcinoma (AC) are the most common histological types, with AC patients having worse prognosis. Over the last two decades, incidence rates of AC have increased, highlighting the importance of further understanding AC tumorigenesis, and the need to investigate new treatment options. The cytokine TGF-β functions as a tumour suppressor in healthy tissue. However, in tumour cells this suppressive function can be overcome. Therefore there is an increasing interest in using TGF-β inhibitors in the treatment of cancer. Here, we hypothesize that TGF-β plays a different role in SCC and AC. Analysis of RNA-seq data from the TCGA, using a TGF-β response signature, resulted in separate clustering of the two subtypes. We further investigated the expression of TGF-β-signalling related proteins (TβR1/2, SMAD4, pSMAD2, PAI-1, αvβ6 and MMP2/9) in a cohort of 62 AC patients. Low TβR2 and SMAD4 expression was associated with worse survival in AC patients and interestingly, high PAI-1 and αvβ6 expression was also correlated with worse survival. Similar correlations of TβR2, PAI-1 and αvβ6 with clinical parameters were found in previously reported SCC analyses. However, when comparing expression levels between SCC and AC patient samples, pSMAD2, SMAD4, PAI-1 and αvβ6 showed lower expression in AC compared to SCC. Because of the low expression of core TβR1/2, (p-)SMAD2 and SMAD4 proteins and the correlation with worse prognosis, TGF-β pathway most likely leads to tumour inhibitory effects in AC and therefore the use of TGF-β inhibitors would not be recommended. However, given the correlation of PAI-1 and αvβ6 with poor prognosis, the use of TGF- β inhibitors might be of interest in SCC and in the subsets of AC patients with high expression of these TGF-β associated proteins.

## Introduction

Cervical cancer is the fourth most common cancer in women worldwide (1). It is caused by a persistent infection with high risk human papillomavirus (HPV) (2). Tumour progression is facilitated by the failing control of the host’s immune response, combined with several immune escape mechanisms of the virally infected cells (3).

Histologically, cervical cancer can be classified as squamous cell carcinoma (SCC), adenocarcinoma (AC), adenosquamous carcinoma (ASC), and rare variants (4), each accounting for approximately 70-75%, 20-25%, 3-5%, and <1% of the cases, respectively (5–8). In contrast to SCC, the absolute and relative incidence rates of cervical AC have increased over the last two decades, predominantly in young women, and in developed countries (6, 8–12). When compared to SCC, AC histology was associated with higher recurrence rates, worse prognosis and survival, showed different dissemination patterns, and a different response to similar treatment regimens (11, 13). Furthermore, we and others have shown that the AC subtype represents a different spectrum of oncogenic mutations and has a different immunological and genomic pathway activation profile, compared to SCC (14–21).

Transforming growth factor-β (TGF-β) is a multifunctional cytokine that functions as a tumour suppressor in healthy cells, by being a potent inducer of growth arrest and apoptosis, maintaining tissue (microenvironment) homeostasis (22). In malignancy, tumour cells can overcome the cytostatic effects of TGF‐β signalling, mostly by acquiring mutations in the canonical signalling components (eliminating the tumour suppressor function) or by adaptation of TGF‐β signalling through non‐canonical signalling (leading to tumour promoting effects) (22–24). Accordingly, interest in TGF-β counteracting immunotherapies is gaining momentum (25), (26). 

In normal tissue homeostasis, TGF-β is secreted into the extracellular matrix as part of a latent complex. TGF-β activators, such as integrins and proteases, release TGF-β from its latent state. Once activated, TGF-β initiates signalling by binding TGF-β receptor 2 (TβR2), which then forms a heteromeric complex with TGFβ receptor 1 (TβR1). TβR2 phosphorylates TβR1, which subsequently phosphorylates Sma and Mad related protein 2 (SMAD2) or SMAD3. These SMADs form heteromeric complexes with common mediator SMAD4, allowing for translocation into the nucleus. There, they interact with various transcription factors to regulate transcription of genes such as plasminogen activator inhibitor 1 *(PAI-1)*, *SMAD7*, matrix metalloproteinases *(MMP’s), collagen type I*, and *fibronectin* (27, 28).

The TGF-β signalling pathway has been investigated predominantly in cervical cancer cell lines and in SCC histology (29–35). Previous studies of our group found that TGF-β1 expression in SCC patients was strongly associated with tumour infiltration within surrounding stroma and thus poor outcome, and TGF-β1 expression positively correlated with PAI-1 expression (31, 33). PAI-1 expression levels correlated with poor overall and disease free survival, while high SMAD4 expression was related to low infiltration depth (33, 34). A potential difference in TGF-β signalling between SCC and AC subtypes was suggested in these studies. However, too few AC patients were included to allow for a meaningful comparison.

Research concerning TGF-β signalling in cervical AC is scarce. With the rising incidence rates of cervical AC, it is important to further distinguish the biological and immunological processes that drive the tumorigenesis of this histological subtype, as this knowledge will contribute to the development of more precise, tumour-specific treatment approaches. Here, we hypothesised that the TGF-β pathway plays a different role in cervical AC compared to SCC (19, 33).

The aim of this study was to investigate the TGF-β pathway in cervical AC. We first analysed RNA-seq data from the TCGA, using a TGF-β response signature. Subsequently, we systematically investigated the protein expression of core canonical pathway members (SMAD4, pSMAD2, TβR1 and TβR2) and TGF-β pathway regulators and transcriptional targets (PAI-1, alpha-v beta-6 integrin (αvβ6 integrin), MMP2 and MMP9), and determined associations between protein expression and clinicopathological parameters in a well-defined, consecutive cohort of cervical AC patients.

## Material and Methods

### Patients and Ethical Statement

This is a retrospective, single-centre- cohort study. All human tissues were used according to the Code of Conduct for responsible use of human tissues in the context of health research 2011 (https://www.bbmri.nl/sites/bbmri/files/styles/Federa_code_of_conduct_english.pdf). (All women included, were diagnosed with cervical AC, International Federation of Gynaecology and Obstetrics (FIGO) stage 1b-2a, and underwent a radical hysterectomy with lymphadenectomy as primary treatment at the Leiden University Medical Center (LUMC) between January 1990 and December 2005. Patients of whom sufficient representative tumour material, preserved in formalin-fixed, paraffin-embedded tissue (FFPE) blocks, was available in the archives of the department of Pathology of the LUMC were included. Clinical files were reviewed to collect data including age, FIGO stage, tumour diameter, invasion depth, lymph-vascular space infiltration (LVSI), parametrial involvement, tumour positivity of the surgical resection margins, tumour positivity of the pelvic lymph nodes, adjuvant treatment, disease recurrence and survival.

Conventional histology sections were stained with haematoxylin and eosin, and reviewed by an experienced pathologist to select only the ‘usual type’ endocervical ACs, according to the World Health Organization (WHO) classification of tumours (4). All tumours were typed for HPV, previously (18).

### Immunohistochemistry

Immunohistochemistry was performed to investigate expression of TβR1 and TβR2, pSMAD2, SMAD4, PAI-1, αvβ6, MMP2 (gelatinase-A) and MMP9 (gelatinase-B). The primary antibodies used are listed in **Supplementary Table 1**.

Immunohistochemistry was performed and evaluated essentially as described before (29, 31, 32, 36). See Supplementary Methods for further details. To compare AC and SCC, immunohistochemistry data from SCC performed in previous studies was used, in which immunohistochemistry and evaluation was essentially as described in this study (29, 32, 33, 37). As a correction for comparing immunohistochemistry stainings, 3-5 SCC cases were taken along in the current AC immunohistochemistry staining.

### Bioinformatical Analysis

RNA-seq and clinical data of AC and SCC were obtained from TCGA. The core set described in (19), was used for bioinformatical analysis. The most optimal number of groups within the TCGA data, was based on the silhouette value where a higher value indicates a better underlying number of groups. Overall similarities between AC and SCC, were visualized based on multidimensional scaling and the heatmap was based on scaled expression data and coloured by AC or SCC status.

### Statistics

Statistical analysis was performed with IBM Statistical Package for the Social Sciences (SPSS) Statistics (version 23, IBM corp., Chicago, IL, USA). Data were processed using the Chi-square test or Fisher’s exact test for categorical variables, Student’s t-test for parametric continuous variables, or one-way analysis of variance for numerical data when comparing more than two groups. Correlation was tested by the Spearman rho correlation coefficient in non-parametric data and the Pearson correlation in cases of normality. Kaplan Meier survival curves and the Log Rank test were used to determine between-group differences in disease-specific survival (DSS) and recurrence-free survival (RFS). Multivariate analysis was performed using a Cox proportional hazard model with stepwise regression. For all reported tests, *P* values were two-sided and *P* values <0.05 were considered to indicate statistical significance.

## Results

### TCGA Analyses

**Figure 1:**
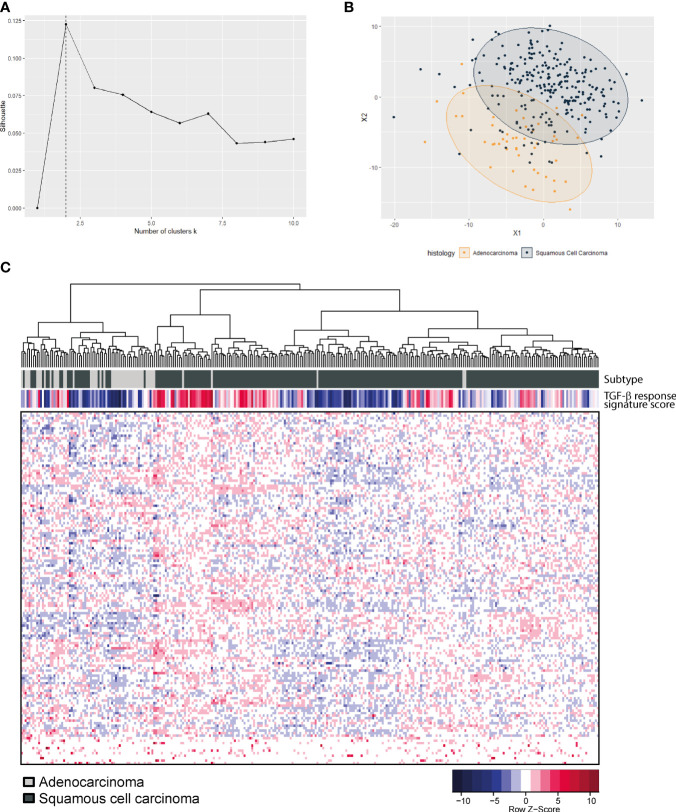
*TGF-β pathway gene expression in the TCGA cervical cancer dataset.* **(A)** Optimal amount of clusters was calculated based on the silhouette value and was found to be two. **(B)** Multidimensional scaling shows a separate clustering of AC (yellow) and SCC (dark blue). **(C)** Heatmap showing unsupervised hierarchical clustering analysis of 144 SCC (dark grey) and 31 AC (light grey) patient samples on the X-axis and 153-gene TGF-β response signature genes on the Y-axis.

To determine if the TGF-β pathway plays a different role in AC versus SCC, expression data from TCGA on cervical cancer were analyzed (19). In this dataset, 144 squamous cell carcinomas and 31 adenocarcinomas were present. We determined the expression of a 153-gene TGF-β response signature (38) (**Supplementary Table 2**) in every tumor, and used these data to analyze the clustering of AC and SCC patients. The optimal amount of clusters in this data was two, based on the silhouette value (**Figure 1A**). Multidimensional scaling (**Figure 1B**) showed the clustering into two major subclasses; subclass A, presenting mostly AC samples (yellow), and subclass B, presenting mostly SCC patients (dark blue). Unsupervised hierarchical clustering of both the patient samples and signature genes, resulted in a heatmap confirming clustering of the SCC samples (dark gray) separately from the AC samples (light gray) (**Figure 1C**). As AC and SCC clustered mostly separately based on the TGF-β response signature, this indeed confirmed our hypothesis that AC is different from SCC in their response to TGF-β. We investigated this further at the protein level.

### Patients

**Figure 2:**
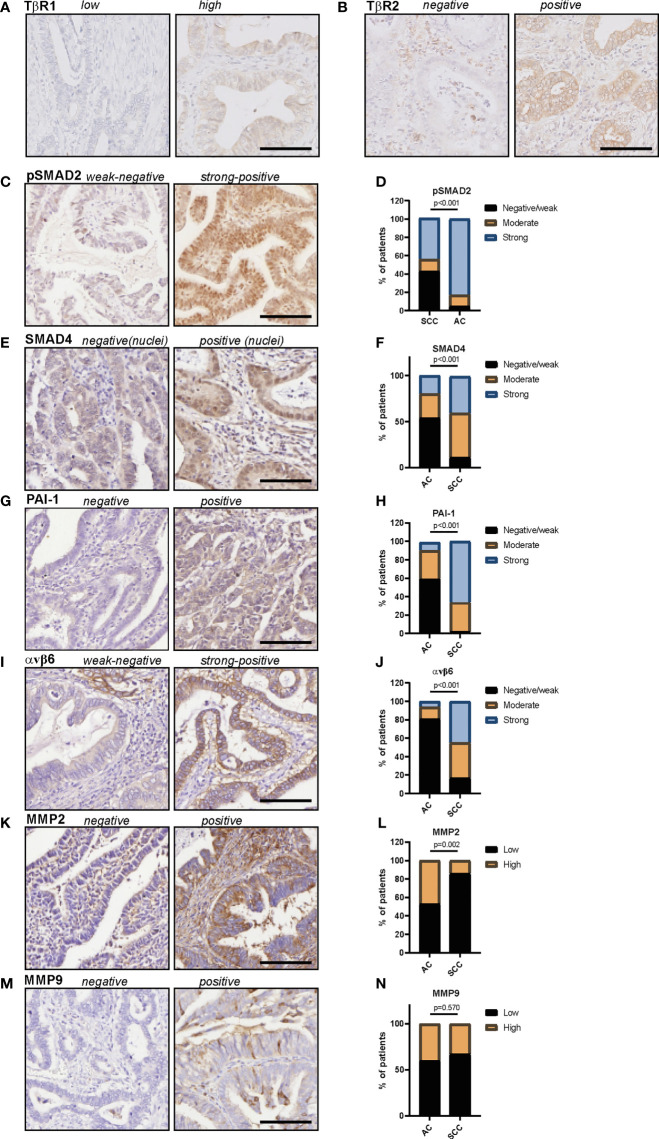
*Protein expression of TβR1, TβR2, pSMAD2, SMAD4, PAI-1, αvβ6 and MMP2 and MMP9 in cervical adenocarcinomas.* **(A)** example of *TβR1* staining, **(B)** example of *TβR2* staining, **(C)** example of pSMAD2 staining, **(D)** pSMAD2 staining in AC and SCC samples. **(E)** Example of SMAD4 staining, **(F)** SMAD4 staining in AC and SCC samples. **(G)** Example of PAI-1 staining, **(H)** PAI-1 staining in AC and SCC samples. **(I)** Example of αvβ6 staining, **(J)** αvβ6 staining in AC and SCC samples. **(K)** Example of MMP2 staining, **(L)** MMP2 staining in AC and SCC samples. **(M)** Example of MMP9 staining, **(N)** MMP9 staining in AC and SCC samples. All protein expression scores can be found in **Table 2** (AC) and **Table 4** (SCC and AC). P values are calculated using Chi-squared test. AC, adenocarcinoma; SCC, squamous cell carcinoma. Scale bar represents 100 μm.

**Figure 3:**
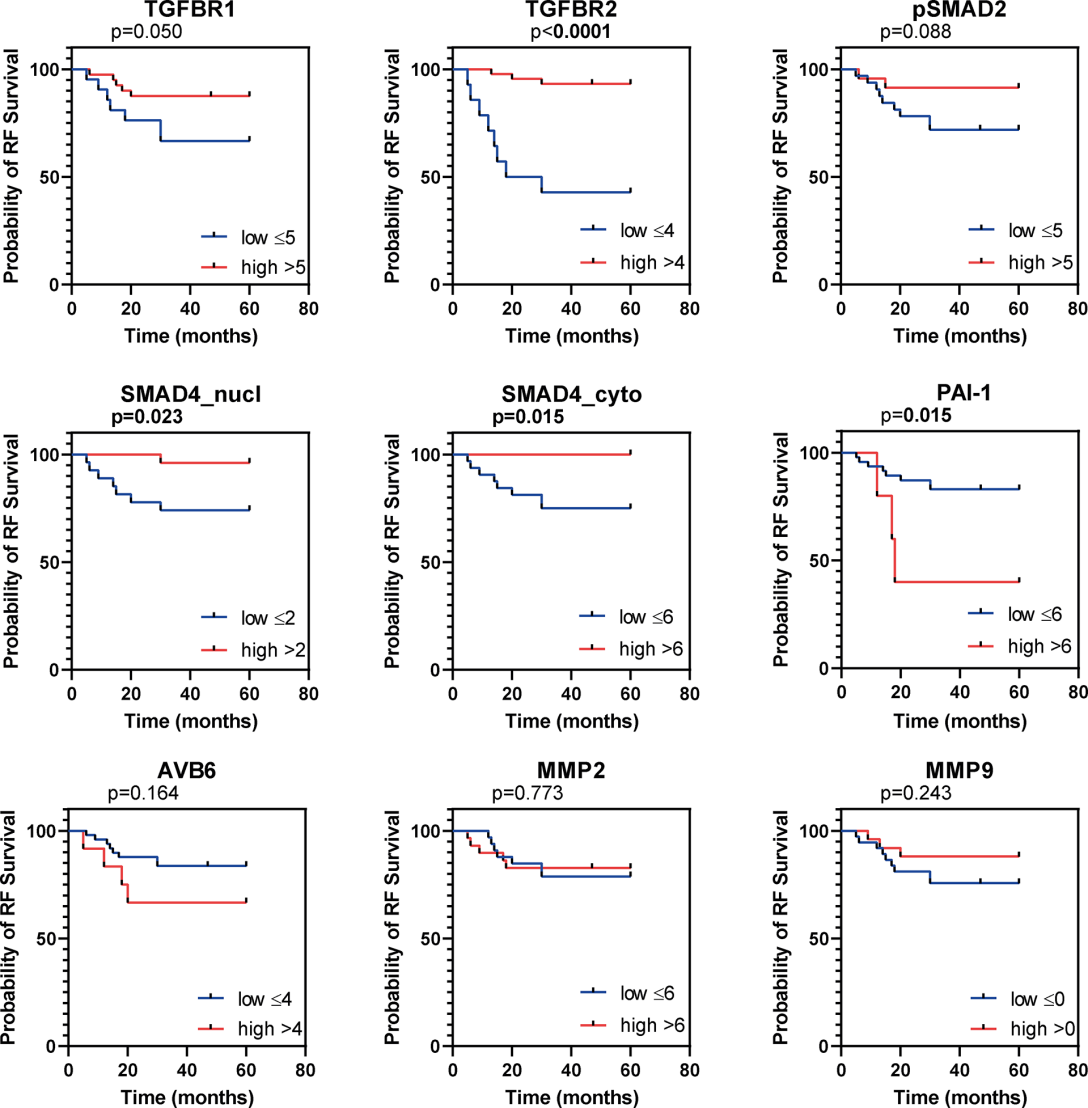
*Kaplan Meier curves for recurrence free survival (RFS).* Kaplan Meier curves for RFS of the institutional AC cohort, dichotomized as indicated by the numbers in the legend. P values based on log-rank test, bold indicates significance (p ≤ 0.05).

**Figure 4:**
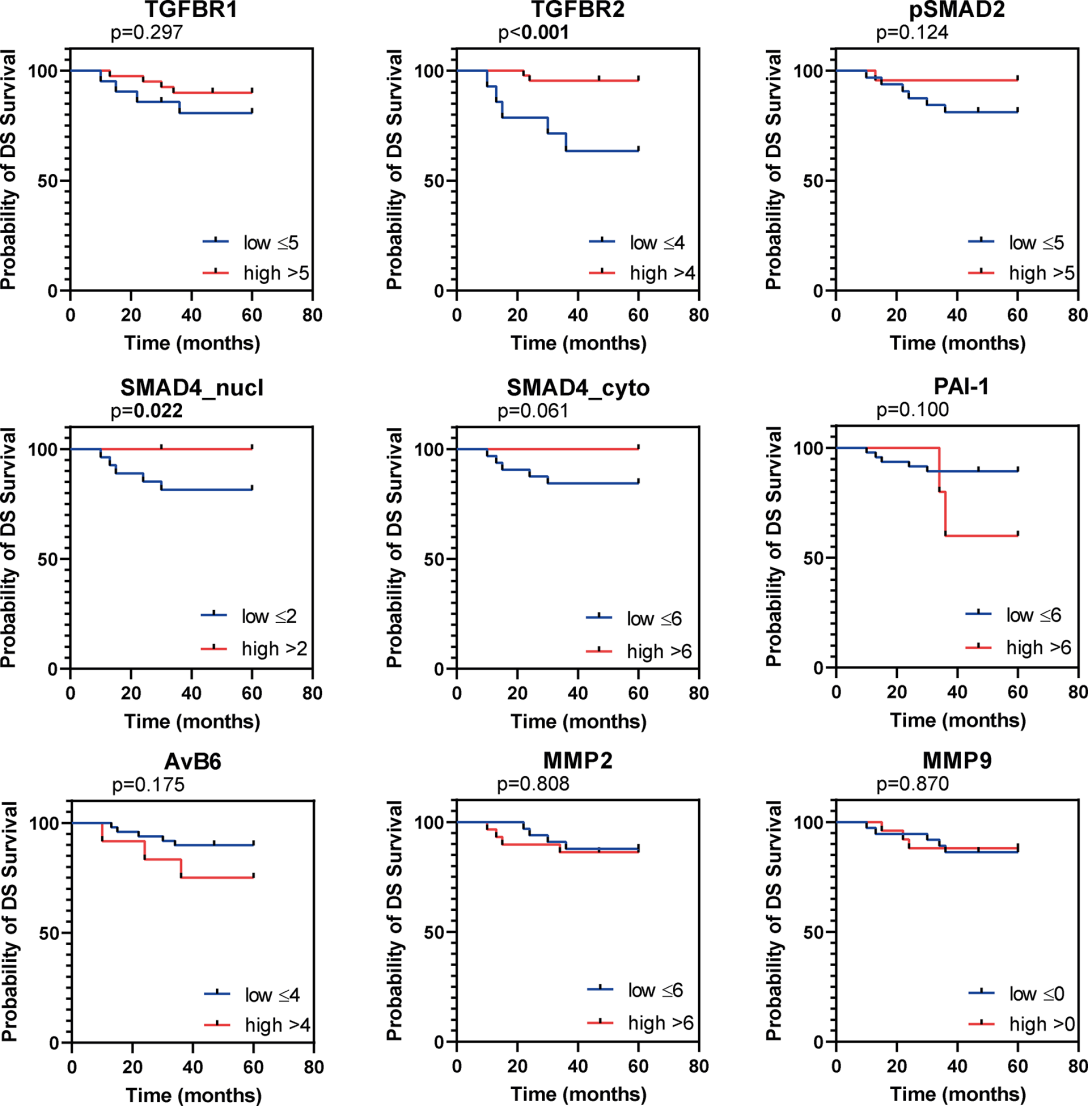
*Kaplan Meier figures for disease specific survival (DSS).* Kaplan Meier curves for DSS of the institutional AC cohort, dichotomized as indicated by the numbers in the legend. P values based on log-rank test, bold indicates significance (p ≤ 0.05).

In total 62 patients with usual type cervical adenocarcinoma were included in this study. Clinicopathological characteristics are summarized in **Table 1**. Protein expression scores are summarized in **Table 2** and **Figure 2**, examples of stainings are shown in **Figure 2**. Univariate survival analysis is shown in **Table 3** and illustrative Kaplan Meier Curves for RFS and DSS are shown in **Figure 3** and 4. A comparison of protein expression scores between this cohort of 62 ACs and a previously analysed and reported cohort of SCCs (29, 32, 33, 37) is summarized in **Table 4** and shown in graphs in **Figure 2**.

**Table 1:** Baseline characteristics of the institutional AC cohort.

Baseline characteristics	*n*=62	
Age in years, median (IQR)	41	(34,0-48,3)
FIGO stage 1b, n (%)	59	(95)
FIGO stage 2a	3	(5)
Tumour size in mm, median (IQR)	25	(16,5-34,3)
Infiltration depth in mm, median (IQR)	11	(5,0-14,8)
LVSI, n (%)*	14	(29)
Parametrium tumour positive, n (%)	1	(2)
Pelvic lymph nodes tumour positive, n (%)	10	(16)
Resection margins tumour positive, n (%)	13	(21)
HPV positive, n (%)	53	(85)
Multiple infection, n (%)**	3	(5)
HPV 16, n (%)	28	(45)
HPV 18, n (%)	23	(37)
HPV 45, n (%)	3	(5)
Adjuvant radiation, n (%)***	20	(32)
Adjuvant chemoradiation, n (%)***	3	(5)
Follow-up in months, median (IQR)	156	(82-205)
Recurrent disease, n (%)	13	(21)
Locoregional recurrence, n (%)	9	(15)
Distant metastasis, n (%)	4	(6)
Overall death, n (%)	20	(32)
Disease specific death, n (%)	12	(19)

Baseline characteristics. Baseline characteristics and clinicopathological parameters of all included cervical adenocarcinoma patients. *Of 14 cases the LVSI status was unknown. **Two cases were HPV16 and HPV18 positive, one case was HPV18 and HPV31 positive. ***Adjuvant treatment was proposed in case of tumour positive lymph nodes, tumour positive resection margins, tumour positive parametria, or in case of two or more of the following three unfavourable factors: tumour size >= 40mm, infiltration depth >=15mm, LVSI.

IQR, interquartile range; FIGO, International Federation of Gynaecology and Obstetrics; LVSI, lymph vascular space infiltration; HPV, human papillomavirus.

**Table 2:** Protein expression scores.

	Protein:	TβR1	TβR2	pSMAD2	SMAD4 (nuclear)	SMAD4 (cytoplasm)	PAI-1	αvβ6	MMP2	MMP9
		*n*=60	*n*=58	*n*=55	*n*=53	*n*=53	*n*=52	*n*=61	*n*=62	*n*=62
Percentage	<1%	0 (0)	5 (9)	1 (2)	17 (32)	7 (13)	6 (12)	13 (21)	0 (0)	37 (60)
	1-5%	5 (8)	1 (2)	11 (20)	11 (21)	2 (4)	12 (23)	18 (29)	4 (7)	17 (27)
	6-25%	2 (4)	5 (9)	12 (22)	14 (26)	4 (8)	7 (13)	17 (28)	9 (15)	8 (13)
	26-50%	5 (8)	6 (10)	7 (13)	8 (15)	12 (23)	7 (13)	4 (7)	6 (10)	0 (0)
	51-75%	9 (15)	16 (27)	6 (11)	1 (2)	4 (8)	14 (27)	4 (7)	17 (27)	0 (0)
	>75%	39 (65)	25 (43)	18 (33)	2 (4)	24 (45)	6 (12)	5 (8)	26 (42)	0 (0)
Intensity	Negative	0 (0)	5 (9)	1 (2)	17 (32)	7 (13)	6 (12)	13 (21)	0 (0)	37 (60)
	Dull	28 (47)	18 (31)	18 (33)	19 (36)	21 (40)	33 (63)	26 (43)	12 (19)	16 (26)
	Clear	32 (53)	28 (48)	27 (49)	17 (32)	20 (38)	13 (25)	18 (29)	29 (47)	9 (15)
	Intense	0 (0)	7 (12)	9 (16)	0 (0)	5 (9)	0 (0)	4 (7)	21 (34)	0 (0)
Total score	Negative (0)	0 (0)	5 (9)	1 (2)	17 (32)	7 (13)	6 (11)	13 (21)	0 (0)	37 (60)
	Weak (2-4)	11 (18)	10 (17)	24 (44)	26 (49)	16 (30)	26 (50)	36 (59)	15 (24)	25 (40)
	Moderate (5-6)	19 (32)	25 (43)	12 (22)	8 (15)	9 (17)	15 (29)	8 (13)	18 (29)	0 (0)
	Strong (7-8)	30 (50)	18 (31)	18 (32)	2 (4)	21 (40)	5 (10)	4 (7)	29 (47)	0 (0)

Protein expression scores. Immunohistochemical assays were evaluated by the scoring system from Ruiter et al (36). Values are presented in number of patients (percentage).

**Table 3:** Univariate survival analysis.

Protein	Expression	*n*	RFS	Hazard Ratio	*p* value	DSS	Hazard Ratio	*p* value
TβR1	low (≤5)	20	65%	0.4 (0.1-1.1)	0.070	70%	0.5 (0.1-1.4)	0.156
	high (>5)	40	85%			85%		
TβR2	low (≤4)	15	46%	0.1 (0.04-0.41)	**<0.001**	53%	0.2 (0.05-0.52)	**0.001**
	high (>4)	43	91%			91%		
pSMAD2	low (≤5)	32	69%	0.3 (0.1-1.2)	0.057	72%	0.3 (0.1-1.3)	0.082
	high (>5)	23	91%			91%		
SMAD4 nucl	low (≤2)	27	74%	0.3 (0.05-1.19)	0.060	74%	0.1 (0.02-1.01)	**0.021**
	high (>2)	26	92%			96%		
SMAD4 cyto	low (≤6)	32	75%	0.2 (0.02-1.32)	0.053	78%	0.2 (0.02-1.5)	0.079
	high (>6)	21	95%			95%		
PAI-1	low (≤6)	47	83%	4.5 (1.2-17.2)	**0.016**	85%	4.2 (1.1-16.5)	**0.023**
	high (>6)	5	40%			40%		
Avβ6	low (≤4)	49	84%	2.4 (1.0-6.1)	0.055	86%	2.6 (1.1-6.6)	**0.046**
	high (>4)	12	58%			58%		
MMP2	low (≤6)	33	82%	1.0 (0.3-3.0)	0.800	79%	1.2 (0.4-3.6)	0.993
	high (>6)	29	79%			79%		
MMP9	negative (0)	37	76%	0.6 (0.2-2.0)	0.407	78%	0.8 (0.2-2.2)	0.517
	positive (>0)	25	84%			84%		

Univariate survival analysis. Univariate survival analysis for RFS and DSS for all TGF-β pathway associated proteins analysed in the present study. Values are calculated by Log Rank (Mantel-Cox) test. RFS, recurrence free survival; DSS, disease-free survival. Bold values indicate significance (p < 0.05).

**Table 4:** Protein expression scores; comparison between AC and SCC.

		AC	SCC	*p* value
		*n* (%)	*n* (%)	
pSMAD2	*n*	56	65	**<0.001**
	Weak/neg (<25%)	24 (43)	3 (5)	
	Moderate (25-50%)	7 (13)	8 (12)	
	Strong (>50%)	25 (45)	54 (83)	
SMAD4 (nuclear)	*n*	54	62	**<0.001**
	Weak/neg (<25%)	29 (54)	7 (11)	
	Moderate (25-50%)	14 (26)	30 (48)	
	Strong (>50%)	11 (20)	25 (40)	
PAI-1	*n*	53	85	**<0.001**
	Weak (2-4)	32 (60)	2 (2)	
	Moderate (5-6)	16 (30)	27 (32)	
	Strong (7-8)	5 (9)	56 (66)	
*Avβ6*	*n*	62	64	**<0.001**
	Weak (2-4)	50 (81)	11 (17)	
	Moderate (5-6)	8 (13)	24 (38)	
	Strong (7-8)	4 (6)	29 (45)	
MMP2	*n*	62	21	**0.002**
	Low (<=6)	33 (53)	18 (86)	
	High (>6)	29 (47)	3 (14)	
MMP9	*n*	62	21	0.570
	Low (<=6)	37 (60)	14 (67)	
	High (>6)	25 (40)	7 (33)	

Protein expression scores. A comparison between AC and SCC of protein expression scores of pSMAD2, nuclear SMAD4, PAI-1, αvβ6, MMP2 and MMP9. Values are presented in number of patients (percentage). P-values are calculated using Chi-squared test. Bold value indicates significance (p <0.05). AC, adenocarcinoma; SCC, squamous cell carcinoma.

### TβR1 and TβR2 Expression

All samples presented positive TβR1 expression (**Table 2**). A cytoplasmic staining was more prevalent than membranous staining (**Figure 2A**). TβR1 expression scores were dichotomized based on the 25^th^ percentile of the total score (=5). Low expression was associated with increased tumour size (mean diameter 35 ± 16mm vs. 22 ± 10mm, *p=*0.001). A trend was observed for association between low expression and lymph node positivity (6/20 (30%) vs. 4/39 (10%), *p*=0.056). TβR1 expression was not associated with survival (**Table 3**; **Figures 3**, **4**).

Moderate/strong TβR2 expression was seen in the majority (74%) of cervical ACs (**Table 2**; **Figure 2B**). Low expression was associated with advanced tumour size (35 ± 17mm vs. 23 ± 11mm, *p=*0.002) and recurrent disease [8/15 (53%) vs. 4/43 (9%), *p=*0.001]. Univariate survival analysis showed a worse RFS (HR 0.1, 95% CI 0.04-0.41) and DSS (HR 0.15, 95% CI 0.05-0.52) for low expression (**Table 3**; **Figures 3**, **4**). However, in multivariate survival analysis, TβR2 expression was not an independent predictor for RFS (HR 0.3, 95% CI 0.1-1.6) or for DSS (HR 0.5, 95% CI 0.74-3.0).

### pSMAD2 and SMAD4 Expression

Overall, pSMAD2 and nuclear SMAD4 staining was more weakly expressed in AC compared to SCC (**Table 4**, **Figures 2C–F**). Nuclear pSMAD2 staining was observed in almost all AC tumours (only one tumour was completely negative, **Table 2** and **Figures 2C, D**). Nuclear SMAD4 staining was present in 68% of the tumours, and cytoplasmic SMAD4 staining in 87% of the tumours (**Table 2**; **Figures 2E, F**). For SMAD4, four cases showed weak nuclear staining but absent cytoplasmic staining, whilst 13 cases showed absent nuclear staining but positive cytoplasmic staining. For SMAD4, as well as for pSMAD2, a typical staining pattern was observed with an intense positive nuclear staining on the tumour edges, and a weak/negative staining pattern towards the centre of the tumour fields.

Low pSMAD2 expression was associated with larger tumour size (mean diameter 32 ± 15mm vs. 20 ± 10mm, *p*=0.003), higher invasiveness (mean infiltration depth 14 ± 8mm vs. 8 ± 4mm, *p=*0.001), but pSMAD2 expression was not associated with RFS or DSS (**Table 3**; **Figures 3**, **4**).

Nuclear- nor cytoplasmic SMAD4 expression was associated with any of the clinicopathological parameters. However, univariate survival analysis revealed a trend for worse RFS (HR 0.3, 95% CI 0.05-1.19), and worse DSS (HR 0.1, 95%CI 0.02-1.01) for low nuclear SMAD4 expression and a worse RFS (HR 0.2, 95% CI 0.02-1.32) and DSS (HR 0.2, 95% CI 0.02-1.5) for low cytoplasmic SMAD4 expression (**Table 3**; **Figures 3**, **4**). Multivariate analysis revealed that nuclear SMAD4 expression was an independent predictor for RFS (HR 0.2, 95% CI 0.04-0.99) besides tumour size (HR 1.1, 95% CI 1.01-1.11), but not for DSS (HR 0.1, 95% CI 0.01-1.64). Cytoplasmic SMAD4 expression was not an independent predictor for RFS (HR 0.2, 95% CI 0.02-1.59) or DSS (HR 0.1, 95%CI 0.01-1.11).

### PAI-1 Expression

Weak PAI-1 expression was observed in the majority of cases, and was more weakly expressed in AC compared to SCC (**Table 3**; **Table 4** and **Figures 2G, H**). PAI-1 expression was not associated with the clinicopathological parameters, although a trend association was observed between moderate/strong PAI-1 expression and lymph node metastasis (5/20 (25%) vs. 2/32 (6%), *p=*0.054).

Univariate survival analysis revealed the worst RFS and DSS for patients with high PAI-1 expression (**Table 3**; **Figures 3**, **4**). In multivariate analyses for RFS and DSS, PAI-1 expression was not an independent predictor for survival (HR 3.2, 95%CI 0.6-16.3, and HR 3.1, 95%CI 0.6-15.6, respectively).

### Avβ6 Expression

Overall, αvβ6 was weakly expressed in AC compared to SCC (**Table 4**; **Figures 2I, J**). The characteristic αvβ6 staining of intense staining at peripheral tumour borders and weaker staining centrally within the tumour nest was less obvious in AC compared to SCC, and was only found in larger, undifferentiated AC samples (32).

High αvβ6 expression was associated with tumour size (mean tumour size 34 ± 18mm for high expression vs. 25 ± 13mm for low expression, *p=*0.049). Univariate survival analysis revealed a worse RFS and DSS for high αvβ6 expression (**Table 3**; **Figures 3**, **4**). In multivariate analysis αvβ6 expression was not an independent predictor for RFS (HR 2.1, 95% CI 0.6-7.3) or DSS (HR 2.1, 95% CI 0.6-7.2).

### MMP2 and MMP9 Expression

Moderate to strong MMP2 expression was shown in 76% of the cases (**Table 2**; **Figures 2K, L**). The typical tumour border staining [as previously reported in SCC (37)] was less obvious in AC. Although a stronger staining pattern on the edges of the tumour outline was observed in 20 tumours (32%) versus homogenous staining in all other tumours.

Low MMP2 expression was associated with deeper tumour infiltration (mean infiltration depth 13 ± 8mm vs. 9 ± 4mm, *p=*0.025), but not with any of the other clinicopathological characteristics. MMP2 expression was not associated with RFS or DSS (**Table 3**; **Figure 3**, **4**). High MMP2 expression occurred more frequently in AC compared to SCC (**Table 4**; **Figure 2L**).

Weak MMP9 expression was observed in 40% of the tumours, whilst 60% stained negative (**Table 2**; **Figures 2M, N**). MMP9 expression was also seen in different amounts at the tumour stroma near the tumour border and scored as abundant (n=15, 24%), sporadic (n=34, 55%), or negative (n=13, 21%). Neither MMP9 expression in tumour cells, nor MMP9 stromal expression was associated with any of the clinicopathological characteristics, and there were no associations with survival (**Table 3**; **Figures 3** and **4**). No difference in MMP9 expression intensity was found between AC and SCC (**Table 4**; **Figure 2N**).

### TβR2_null_ and SMAD4_null_ Analysis

**Figure 5:**
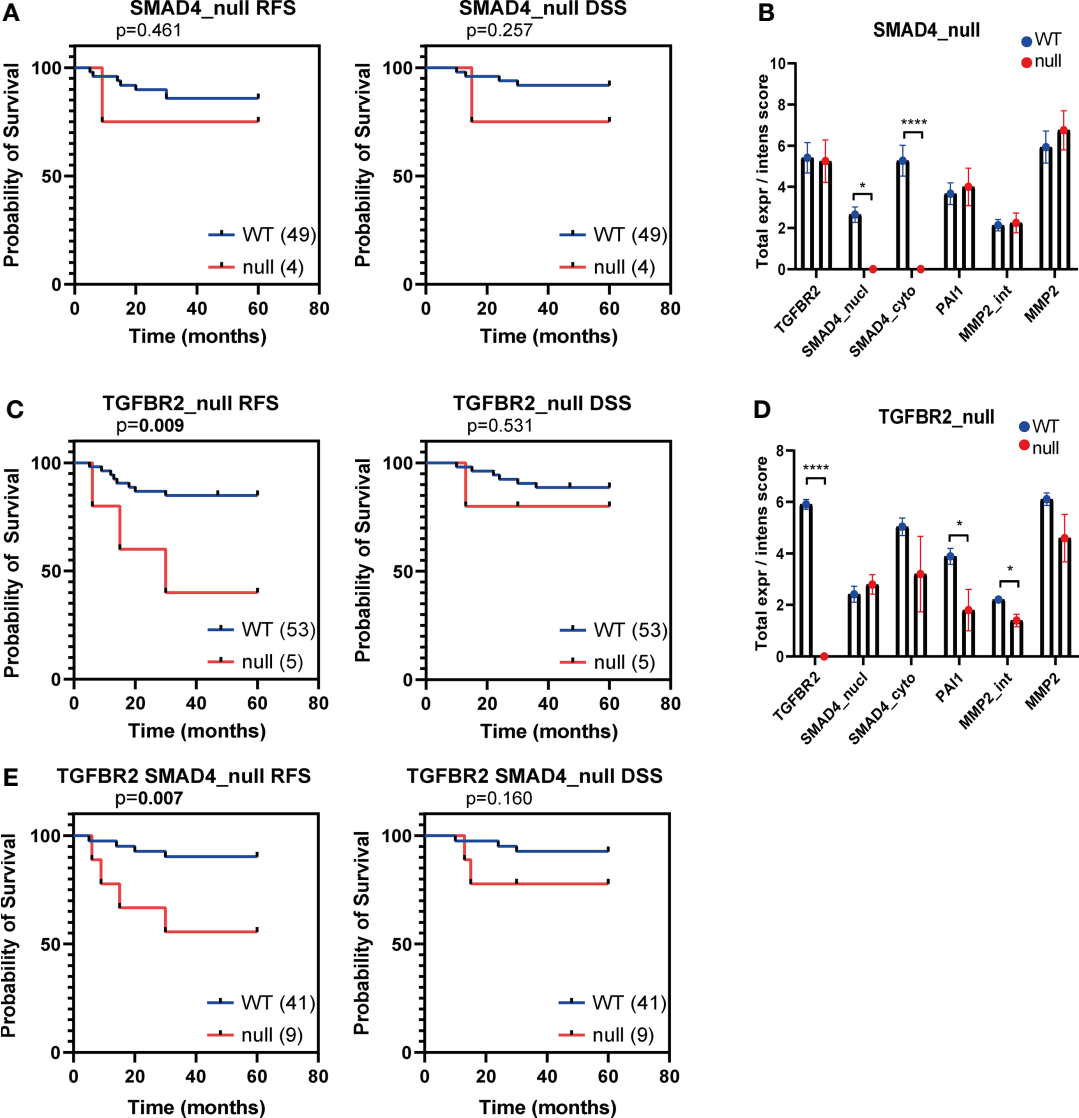
Kaplan Meier curves for recurrence free survival (RFS) and disease specific survival (DSS) for SMAD4_null_ and TβR2 _null_ AC patients samples. Kaplan Meier curves for RFS and DSS of the institutional AC cohort, dichotomized based on SMAD4_null_ **(A)**, TβR2_null_ **(C)**, TβR2_null_ or SMAD4_null_ **(E)** expression. The number of patient samples included in each group is indicated between brackets in the legend. P values based on log-rank test, bold indicates significance (p ≤ 0.05). Protein expression of TβR2, SMAD4 (nuclear and cytoplasmic), PAI-1 and MMP2 were compared in TβR2 wild type versus null **(B)** and SMAD4 wild type versus null **(D)**. Significance is indicated with p<0.05 (*) and p<0.0001 (****).

Previously, the TCGA research network identified a high number of aberrations in tumour suppressor genes related to the TGF-β pathway in both SCC and AC (19). In SCC, both *SMAD4* and *TGFBR2* were mutated in 4% and 8% of the cases, respectively, whereas in AC only *SMAD4* and not *TGFBR2* mutations were observed in 12% of cases. Mutations in these genes are expected to result in absence of or aberrant protein expression. Hence, we assessed the absence of TβR2 and SMAD4 expression in our AC dataset. Indeed, SMAD4 was completely absent (SMAD4_null_, for both nuclear and cytoplasmic staining) in 4/52 AC cases (7.54%) (**Figures 5A, B**). Unexpectedly, also TβR2 expression was absent (TβR2_null_) in 5/58 (8.62%) AC cases (**Figure 5C**). PAI-1 expression and MMP2 expression were both significantly reduced in TβR2_null_ patients (**Figure 5D**). Kaplan Meier survival analysis indicated that the TβR2_null_ group and the TβR2_null_ or SMAD4 _null_ group had a significantly worse RFS compared to the wild type group (**Figures 5C, D** and **E**).

## Discussion

Research concerning TGF-β signalling in cervical AC is scarce. Farley et al., studied TGF-β and TβR protein expression in AC (N=7) and its precursor lesions, and suggested that the neoplastic transformation of the endocervix might be related to deregulated TGF-β, and therewith loss of cell cycle control (39). Fan et al., studied TGF-β protein expression in 66 AC cases (who all received chemo- and immunotherapy prior to surgery), and described a positive expression being an independent predictor for worse survival (40). Previous studies of our group concerning the TGF-β pathway in cervical cancer demonstrated differences in TGF-β pathway activity comparing AC to SCC, however, too few AC samples were included to lead to meaningful conclusions (33). By comparing the expression of a 153-gene TGF-β response signature in AC and SCC patient samples from the TCGA dataset (19), we identified that most AC and SCC cluster separately. This suggests that the TGF-β pathway might indeed play a different role in the tumorigenesis of these cervical carcinoma subtypes. To better characterize cervical AC, we performed a comprehensive IHC analysis of various proteins of the TGF-β canonical pathway and downstream TGF-β pathway targets in a well-defined, consecutive cohort of 62 cervical AC patients. The AC series of our study was carefully classified using an additional mucus staining (14).

**Figure 6:**
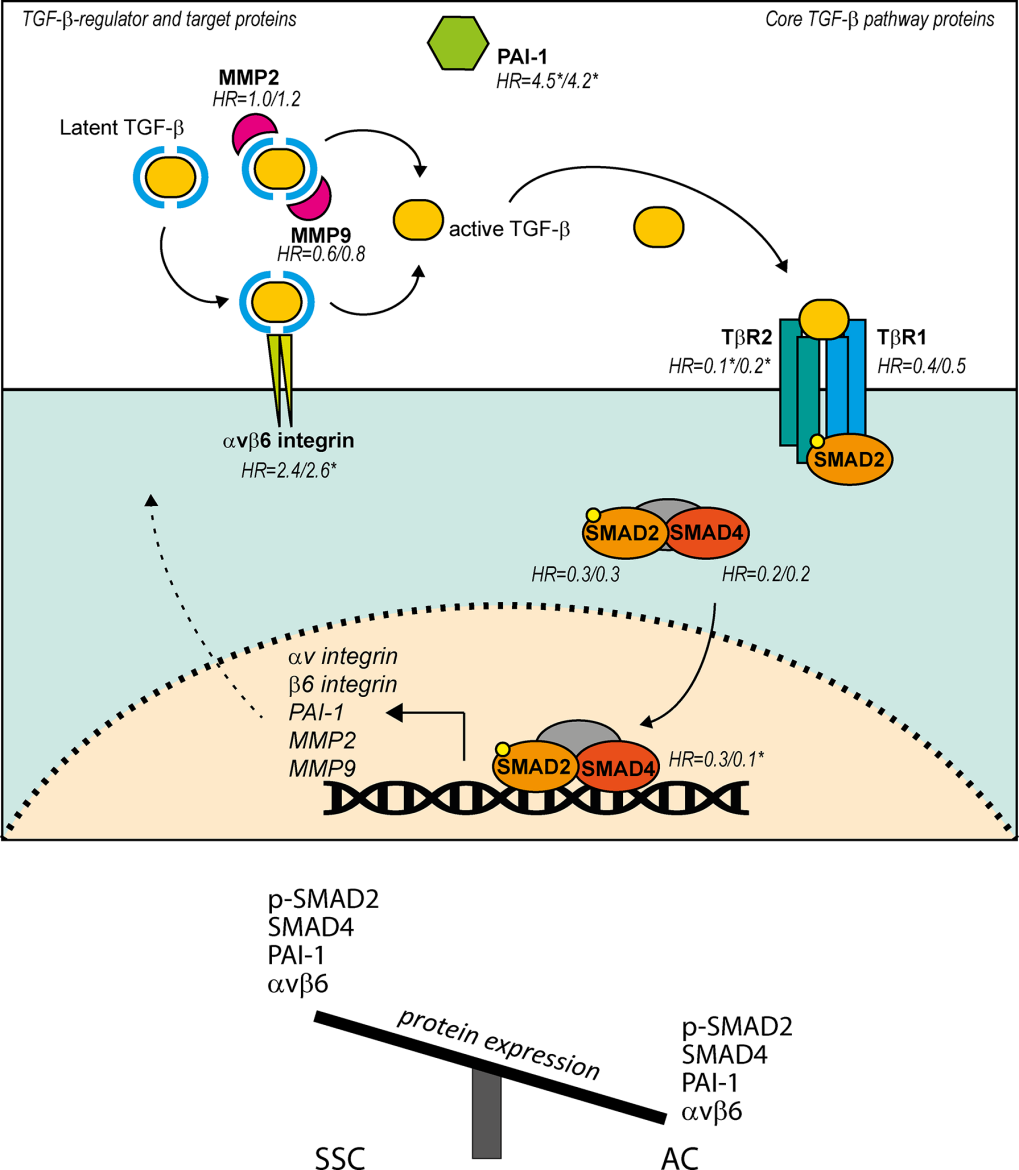
Summary of the presented findings of TGF-β pathway, TGF-β regulator and TGF-β target proteins in AC, and differences in expression between AC and SCC in regard to these proteins. TGF-β pathway starts with binding of TGF-β to TβR2, leading to phosphorylation of TβR1 and subsequent phosphorylation of SMAD2. SMAD2 together with SMAD4 and SMAD3 (not indicated) form a complex and translocate to the nucleus to regulate transcription of TGF-β target genes *PAI-1*, *αv integrin*, *β6 integrin*, *MMP2* and *MMP9*. Avβ6 integrin and MMP2/9 mediate cleavage of latent TGF-β. Hazard ratios (HR) are calculated low over high, and are shown for the proteins investigated in the present study. HRs are shown as RFS/DSS, significance (p ≤ 0.05) is indicated with*.

First, we assessed the expression of the core TGF-β canonical pathway proteins: TGF-β receptors and SMAD proteins. These proteins, are crucial in the signalling cascade to transfer the extracellular signal from activated TGF-β into transcription of target genes, including *PAI-1*, *αvβ6 integrin*, *MMP*’s and several cell cycle inhibitors (**Figure 6**). We showed that low expression of the core TGF-β pathway proteins (TβR1, TβR2, pSMAD2 and SMAD4) was associated with a poor prognosis in AC. Interestingly, a similar correlation to disease progression was observed in SCC (29). Previously, our group reported that all cervical SCC tumours show nuclear pSMAD2 expression and cytoplasmic SMAD4 expression (29). In comparison with SCC, here, pSMAD2 and SMAD4 staining was found to be lower in AC (**Table 4**; **Figures 2D, F**). Overall, the association of low expression of pSMAD2 and SMAD4 with poor prognosis, suggests that inactivation of the TGF-β pathway in AC might result in hampering cytostatic effects of the pathway.

In a few samples, we observed a complete lack of staining for either TβR2 or SMAD4. These TβR2_null_ and SMAD4_null_ cases were identified in 9% and 8% of our cohort, respectively. In the TCGA data, approximately 12% of AC patients showed a mutation or DNA methylation of *SMAD4* (19). This could fit with our findings; the slightly higher percentage might be due to the low sample size in the TCGA dataset (31 AC samples) and of the group deficient in TβR2. In contrast, the TCGA analysis revealed no mutations or DNA methylation for *TGFBR2*, whereas our results suggest that about 9% of the cases had a complete lack of expression. Again, the small AC sample size in the TCGA cohort, and the small sample size of patients deficient in TβR2, might be the cause. Alternatively, this might be the result of epigenetic or posttranslational silencing. Future research should address these questions.

Next, we investigated TGF-β regulating and target proteins αvβ6 integrin and PAI-1. Avβ6 is a transmembrane cell surface receptor that mediates cell adhesion. It binds and activates latent TGF-β (41). In turn, TGF-β upregulates the expression of αvβ6 integrin on human keratinocytes (42). The serine protease inhibitor PAI-1, regulates the cleavage of plasminogen into plasmin by inhibiting the urokinase- and tissue plasminogen activators (uPA and tPA), which is an important mechanism in the regulation, formation, and degradation of the extracellular matrix (ECM) (33, 43). In normal cervical epithelium, PAI-1 and αvβ6 are not- or weakly expressed, while their expression in cervical intra-epithelial neoplasia is strong (32). Interestingly, in AC we observed predominantly weak PAI-1 and αvβ6 staining. This was in contrast with SCC, where PAI-1 and αvβ6 were strongly expressed (**Table 4**; **Figures 2H, J**). Moreover, in AC the characteristic staining pattern of αvβ6 on the tumour-stromal interface was less obvious compared to SCC (32). Despite the overall weak staining, high PAI-1 and αvβ6 expression were associated with a worse outcome. These findings were opposite to the expression of core TGF-β pathway members (TβR and SMAD proteins), suggesting these proteins might be induced by other signalling pathways associated with worse survival outcome (e.g. TNF-α/NFkB) (44). Indeed, neither PAI-1 nor αvβ6 showed correlative (inverse) expression with one of the core TGF-β pathway proteins in AC. This was in contrast to SCC, where TGF-β1 expression inversely correlated with PAI-1 and αvβ6 (31, 32). Thus, although in both AC and SCC high PAI-1 expression correlates with worse outcome, the expression pattern and staining intensity of PAI-1 and of αvβ6 was different between the two subtypes.

In AC, TGF-β expression was found to be higher compared to SCC (33, 45). If TGF-β1 expression is high in AC, but the core pathway proteins and target/regulating proteins are lower in tumour cells, this suggests that TGF-β1 might be acting predominantly on the microenvironment in AC rather than on the tumour cells. TGF-β affects many cell types in the microenvironment, including immune cells, and it can promote tumour progression by evasion of the immune system (24). By activating pro-tumorigenic microenvironment cells like M2 macrophages or by inhibiting CD8 T cell activation, TGF-β1 might result in a tumour promoting effect. Future work should address these points.

Similar to the differences observed in protein expression scores for SCC and AC, our bioinformatical analysis showed that SCC and AC samples fell into separate clusters when looking at the TGF-β response signature. This suggests, that these subsets do show differences in TGF-β signalling. Part of these differences could be explained by the lower staining intensity of pSMAD2, SMAD4, PAI-1 and αvβ6 in SCC. The TGF-β pathway-related protein expression differences observed between AC and SCC are summarized in **Figure 6**. Interestingly, 6/31 of AC samples clustered in the predominant SCC cluster, indicating that these 6 AC patients might respond to TGF-β similarly to SCC patients, and might potentially have higher TGF-β related protein expression. Further investigation is necessary to identify the differences in TGF-β response between AC and SCC.

In conclusion, in AC, similar to SCC, PAI-1 and αvβ6 integrin are unfavourable prognostic factors although this may not be TGF-β pathway related - given the inverse correlation in protein expression levels. Moreover, we showed that in AC there is a low core TGF-β pathway protein expression compared to SCC, and low expression is associated with poor patient survival and worse prognosis. As such, the use of TGF-β inhibitors to treat AC patients with moderate to strong staining for core TGF-β pathway proteins, would not be recommended, as tumour cell proliferation might increase. Alternatively, for AC patients with high expression of TGF-β target proteins such as PAI-1 and αvβ6 integrin, as well as for SCC patients, TGF-β inhibitors could be of clinical interest. Few clinical trials using these inhibitors are including patients with cervical cancer, and targeting TGF-β remains challenging (46, 47). The role of TGF-β on the immune system is gaining interest, and there is increasing evidence that TGF-β affects immunotherapy response in cervical cancer as well (26, 48–51). Differences between AC and SCC, should be taken into account in these studies, as immune cell recruitment has been found to differ between the two subtypes (51). Future studies including validation in *in vitro* and *in vivo* models will be necessary to further elucidate the role of TGF-β in cervical AC and SCC. Our results could also be of relevance for other cancer types with this subclassification, such as lung cancer. Based on the here presented data, characterisation of TGF-β related protein expression in individual AC patients will be important upon patient inclusion in future clinical trials with TGF-β inhibitors, as monotherapy or in combination with other therapies.

## Data Availability Statement

Publicly available datasets were analyzed in this study. This data can be found here: TCGA database, discussed in DOI:10.1038/nature21386.

## Author Contributions

VMS carried out the experiments. RCS and MK performed out the bioinformatical analysis. EMS, ESJ, LR and LM analysed data. Study design was done by VMS, and EJ. VMS and CDdK were responsible for recruitment of patients and collecting samples. VMS and DLM wrote the draft manuscript. ESJ, LR, PtD and CDdK revised the manuscript. All authors had final approval of the submitted and published versions.

## Funding

This study was supported by Cancer Genomics Netherlands (CGC.NL) to LR and PtD, and the Louise Vehmeijer stichting Amsterdam to ESJ.

## Conflict of Interest

The authors declare that the research was conducted in the absence of any commercial or financial relationships that could be construed as a potential conflict of interest.

## Publisher’s Note

All claims expressed in this article are solely those of the authors and do not necessarily represent those of their affiliated organizations, or those of the publisher, the editors and the reviewers. Any product that may be evaluated in this article, or claim that may be made by its manufacturer, is not guaranteed or endorsed by the publisher.

## Supplementary Material

Supplementary Table 1
*Overview of the antibodies used in the present study.* Monoclonal and polyclonal antibodies used for the immunohistochemistry assays. The antigen retrieval method, the dilution used, and the supplying companies are listed, as well as the references concerning the studies of our group where the antibodies were described previously.

Supplementary Table 2 The 153-gene TGF-β response signature as defined by Padua et al. (38). The gene names are indicated, as well as their up- or downregulated status in the original signature.

## Abbreviations

AC, cervical adenocarcinoma; ASC, cervical adenosquamous carcinoma; avβ6, alpha-v beta-6 integrin; DSS, Disease specific survival; FIGO, International Federation of Gynaecology and Obstetrics; HPV, Human Papillomavirus; MMP2, matrix metalloproteinase 2; MMP9, matrix metalloproteinase 9; PAI-1, plasminogen activator inhibitor 1; (p-)SMAD, (phosphorylated-) mothers against decapentaplegic homolog family members; RFS, Recurrence free survival; SCC, cervical squamous cell carcinoma; TCGA, The Cancer Genome Atlas; TGF-β, Transforming Growth Factor–beta; TβR1, Transforming growth factor-beta type I receptor; TβR2, Transforming growth factor-beta type 2 receptor.
