# Supplementary material for: Low Transforming Growth Factor-β Pathway Activity in Cervical Adenocarcinomas
Source: Front Oncol. 2022 Jun 8;12:797453. doi: 10.3389/fonc.2022.797453 (PMC9213724; doi:10.3389/fonc.2022.797453)
Supplement: Supplementary Table 1 — Overview of the antibodies used in the present study. Monoclonal and polyclonal antibodies used for the immunohistochemistry assays. The antigen retrieval method, the dilution used, and the supplying companies are listed, as well as the references concerning the studies of our group where the antibodies were described previously. [file Table_1.pdf]

Protein	Antibody Information*	Antigen retrieval	Chromogen reaction	Dilution	Supplier information	Reference
TβR1	Rabbit pcAb	None	DAB-solution	1:25	Santa Cruz Biotechnology, Santa Cruz, CA, USA	31
TβR2	Rabbit pcAb	None	DAB-solution	1:250	Santa Cruz Biotechnology, Santa Cruz, CA, USA	31
pSMAD2	Rabbit mAb, clone 138D4	Citrate	DAKO-DAB+	1:25	Cell Signalling Technology, Beverly, CA, USA	29
SMAD4	Mouse mAb, (B-8) sc-7966	Citrate	DAB-solution	1:200	Santa Cruz Biotechnology, Santa Cruz, CA, USA	29
PAI1	Mouse mAb, no. 3785	None	DAB-solution	1:100	American Diagnostica Inc., Stamford, CT, USA	31
αvβ6	Mouse mAb, clone 6.2A1	Citrate	DAB-solution	1:400	Biogen Idec, Cambridge, MA, USA	32
MMP-2	Mouse mAb, clone VB3	Citrate	DAKO-DAB+	1:50	Thermo Fisher Scientific, Fremont, CA, USA	37
MMP-9	Rabbit pcAB, clone BEA21	Citrate	DAB-solution	1:200	TNO-PG, Leiden, The Netherlands	37

* all antibodies are anti-human
αvβ6, alpha-v beta-6 integrin; MMP2 and MMP9, matrix metalloproteinase2 and 9; PAI1, plasminogen activator 1; pSMAD2, phospho-SMAD2; TβR1 and TβR2, transforming growth factor-beta type I and type II receptor; mAb, monoclonal antibody; pcAb, polyclonal antibody; DAB-solution, 3,3'-diamino-benzidine-tetrahydrochloride in 0.05M Tris-HCl (1:2000) with 15µl H_2O_2 30%; DAKO-DAB+, DAB+ chromogen (K-3468) in DAKO HRP-substrate buffer (K-3468) (1:50)
