# Supplementary material for: Low Transforming Growth Factor-β Pathway Activity in Cervical Adenocarcinomas
Source: Front Oncol. 2022 Jun 8;12:797453. doi: 10.3389/fonc.2022.797453 (PMC9213724; doi:10.3389/fonc.2022.797453)
Supplement: Supplementary file 3 [file DataSheet_1.docx]

***Supplementary Methods***

*Immunohistochemistry*

Immunohistochemistry was performed on 4μm FFPE sections on silane-coated slides (StarFrost®). Sections were deparaffinised and rehydrated using graded concentrations of ethanol to distilled water. Endogenous peroxidise activity was blocked with 0. 3% MeOH/H_2­_O_2_ for 20 min followed by 12 minutes of antigen retrieval in boiling 0.01 M citrate buffer (pH 6.0). Antigen retrieval was performed for the αvβ6, MMP2, MMP9, pSMAD2, and SMAD4 immunoassays, not for PAI-1, TβR1 and TβR2. After two hours cooling down in citrate buffer, slides were washed twice in phosphate-buffered saline (PBS) and incubated overnight at room temperature with the primary antibody diluted in PBS containing 1% bovine serum albumin. Thereafter, sections were washed 3 times 5 minutes in PBS and incubated for 30 minutes at room temperature with BrightVision poly-horseradish peroxidase anti-mouse/rabbit/rat IgG (ImmunoLogic BV, Duiven, The Netherlands). Again, sections were washed 3 times 5 minutes in PBS and incubated with a 0.05 M Tris-HCl buffer (pH 7.6) containing 0.05% of 3,3’-diaminobenzidine-tetrahydrochloride and 0.0018% H_2_O_2_ or, alternatively, a DAKO- 3,3′-Diaminobenzidine (DAB)+ solution, consisting of 2% 3,3’-diaminobenzidine in chromogen solution in imidazole-HCl buffer (pH 7.5) containing hydrogen peroxide (K-3468, DAKO, Heverlee, Belgium) to visualize immune complexes. The reaction was stopped after 10 minutes by rinsing the slides with distilled water and lastly, the tissue sections were counterstained with Mayer’s haematoxylin. Appropriate positive control sections were stained simultaneously and staining of 2 sections excluding primary antibody were used as negative controls.

*Evaluation of immunohistochemistry staining*

Brown cytoplasmic/membranous staining (MMP2, MMP9, PAI-1, αvβ6, TβR1, TβR2), or brown nuclear staining (SMAD4, pSMAD2) indicated positive protein expression. The slides were evaluated by two researchers (VS, ESJ). When scored differently, slides were re-evaluated until consensus was reached. The staining patterns were scored semi quantitatively according to a scoring-system as proposed by Ruiter et al(1). The percentage of positively stained tumour cells was scored 0 to 5 to indicate the presence of positively stained tumour cells as absent (<1%, 0), sporadic (1-5%, 1), local (6-25%, 2), occasional (26-50%, 3), majority (51-75%, 4), or large majority (>75%, 5). The staining intensity of positively stained tumour cells was scored from 0 to 3 to indicate negative (0), dull (1), clear (2) or bright (3) staining intensity. Final scores were calculated by totalling the scores for percentage and intensity, resulting in a score from 0 to 8. A final score of 0 indicated negative expression, 2-4 weak expression, 5-6 moderate expression, and 7-8 strong expression.

1. Ruiter DJ, Ferrier CM, Van Muijen GNP, Henzen-Logmans SC, Kennedy S, Kramer MD, et al. Quality control of immunohistochemical evaluation of tumour-associated plasminogen activators and related components. Eur J Cancer [Internet]. 1998 Aug [cited 2021 May 3];34(9):1334–40. Available from: https://pubmed.ncbi.nlm.nih.gov/9849413/
